# The Association Between Pleural Empyema and Peripheral Arterial Disease in Younger Patients: A Retrospective National Population-Based Cohort Study

**DOI:** 10.3389/fmed.2021.621330

**Published:** 2021-03-19

**Authors:** Tzu-Yuan Wang, Hsin-Hung Chen, Chun-Hung Su, Sheng-Pang Hsu, Chun-Wei Ho, Ming-Chia Hsieh, Cheng-Li Lin, Chia-Hung Kao

**Affiliations:** ^1^Department of Internal Medicine, College of Medicine, China Medical University, Taichung, Taiwan; ^2^Division of Endocrinology and Metabolism, China Medical University Hospital, Taichung, Taiwan; ^3^Division of Endocrinology and Metabolism, Department of Internal Medicine, Asia University Hospital, Taichung, Taiwan; ^4^School of Medicine, Institute of Medicine and Public Health, Chung Shan Medical University, Taichung, Taiwan; ^5^Chung Sheng Clinic, Nantou, Taiwan; ^6^Institute of Medicine, School of Medicine, Chung Shan Medical University, Taichung, Taiwan; ^7^Division of Cardiology, Department of Internal Medicine, Chung Shan Medical University Hospital, Taichung, Taiwan; ^8^Division of Endocrinology and Metabolism, Department of Medicine, Everan Hospital, Taichung, Taiwan; ^9^Intelligent Diabetes Metabolism and Exercise Center, China Medical University Hospital, Taichung, Taiwan; ^10^Graduate Institute of Integrative Medicine, China Medical University, Taichung, Taiwan; ^11^Division of Clinical Nutrition, China Medical University Hospital, Taichung, Taiwan; ^12^Management Office for Health Data, China Medical University Hospital, Taichung, Taiwan; ^13^College of Medicine, China Medical University, Taichung, Taiwan; ^14^Graduate Institute of Biomedical Sciences Science, College of Medicine, China Medical University, Taichung, Taiwan; ^15^Department of Nuclear Medicine and PET Center, China Medical University Hospital, Taichung, Taiwan; ^16^Department of Bioinformatics and Medical Engineering, Asia University, Taichung, Taiwan; ^17^Center of Augmented Intelligence in Healthcare, China Medical University Hospital, Taichung, Taiwan

**Keywords:** pleural empyema, peripheral arterial disease, retrospective cohort study, NHIRD, National Health Insurance Research Database, Cox regression hazard model

## Abstract

**Background:** To investigate the relationship between pleural empyema (PE) and peripheral arterial disease (PAD).

**Methods:** We conducted a retrospective cohort study using data from the National Health Institute Research Database. Univariable and multivariable Cox's proportional hazard regressions were performed to investigate the association between PE and the risk of PAD. Kaplan–Meier method and the differences were assessed using a log-rank test.

**Results:** The overall incidence of PAD was higher in the PE cohort than in the non-PE cohort (2.76 vs. 1.72 per 1,000 person-years) with a crude hazard ratio (HR) of 1.61 [95% confidence interval (CI) = 1.41–1.83]. After adjustment for age, gender, and comorbidities, patients with PE were noted to be associated with an increased risk of PAD compared with those without PE [adjusted HR (aHR) = 1.18, 95% CI = 1.03–1.35]. Regarding the age-specific comparison between the PE and non-PE cohorts, PAD was noted to be significantly high in the ≤ 49 years age group (aHR = 5.34, 95% CI = 2.34–10.1). The incidence of PAD was higher in the first 2 years, with an aHR of 1.35 (95% CI = 1.09–1.68) for patients with PE compared with those without PE.

**Conclusion:** The risk of PAD was higher if patients with PE were younger than 49 years and within the 2-year diagnosis of PE.

## What is Already Known About This Topic?

1. A previous report mentioned the association between PE and vascular diseases.
2. However, the association between PE and PAD is unknown.

## What Does This Article Add?

1. Our analysis determined that patients with PE had an increased risk of PAD.
2. Especially, they were younger than 49 years, of the female sex, and within the 2-year diagnosis of PE.

## Introduction

In the United States, pleural empyema (PE) affects up to 65,000 patients annually, resulting in an estimated cost of 500 million dollars and a 15% mortality rate (1, 2). The etiology of PE varies from benign inflammatory disease to malignancy (3). PE could occur with pneumonia or secondary to thoracic surgery or chest injury and might cause the collection of purulent fluid in the pleural space. One study revealed that more than half of patients with bacterial pneumonia had PE (4–6). PE has various stages, such as exudative, fibrinopurulent, and organized phases, with its entire course lasting approximately 3–6 weeks. Notably, PE treatment included medicines such as antibiotics or surgical interventions for complete drainage of the infected fluid. Over the years, the incidence of PE has increased worldwide despite the availability of efficient therapies. One report mentioned that the rate of PE in adults caused by pneumococcal pneumonia increased from 7.6 to 14.9% (7, 8).

Peripheral arterial disease (PAD) is a lethal systemic disease caused by atherosclerosis resulting in a two- to six-fold increase in both cardiovascular and cerebrovascular diseases. The annual mortality rate of PAD was 4–6% (9, 10). The overall prevalence of PAD in adults was reported to be 12% in America. Notably, an estimated 8–10 million Americans were diagnosed as having PAD (11). Risk factors of PAD included old age, male sex in the younger age group, smoking, diabetes, hypertension, and dyslipidemia. PAD is characterized by an increased risk of coronary and cerebrovascular events, with a 15%−25% rate of carotid artery stenosis and a 30–50% incidence of coronary artery disease (CAD) (12, 13).

A previous report mentioned the association between PE and vascular diseases, such as stroke and aortic aneurysm, or systemic diseases, such as diabetes and kidney disease (14–17). However, the association between PE and PAD is unknown, and our study used the national claims data to investigate the probability of PAD development in patients with PE.

## Methods

### Data Source

We conducted a retrospective cohort study by using data from the Taiwan National Health Institute Research Database (NHIRD), which was released by the Taiwan National Health Research Institutes. The National Health Insurance program is a mandatory universal health insurance program that offers comprehensive medical care coverage to all Taiwan residents (approximately more than 25 million) (18). Nevertheless, to protect the confidentiality of patients, the data on medical claims have been cryptographically scrambled. For this cohort study, we used a subset of the NHIRD, including files of inpatient claims and registry of beneficiaries. Diagnoses were coded based on the International Classification of Diseases, 9th Edition Clinical Modification (ICD-9-CM). This study was approved by the Institutional Review Board of China Medical University (CMUH104-REC2-115-AR4).

### Sampled Participants

This study collected the complete medical information of patients aged > 20 years who were hospitalized for PE (ICD-9-CM codes 510, 510.0, 510.9) between January 1, 2000, and December 31, 2010, but had no previous medical history of PAD (ICD-9-CM codes 440.0, 440.2, 440.3, 440.8, 440.9, 443, 444.0, 444.22, 444.8, 447.8, and 447.9). The dates of the first health care admission for PE were defined as the index dates. We also excluded a history of empyema that was due to cancer. Overall, the PE cohort comprised 31,051 patients. Four non-PE control subjects for each PE case were frequency-matched based on gender, age group (5-year span), and the calendar year of the index date. Control individuals with a history of PAD before the index date, who had a history of empyema that was due to cancer, and who had incomplete medical records were excluded and replaced with another qualified control subject. Finally, 122,669 non-PE control subjects were included in this study.

### Outcome and Comorbidities

Both PE and non-PE cohorts were followed up until PAD development, being censored due to loss to follow-up, or the end of 2011. Baseline comorbidities including diabetes (ICD-9-CM code 250), hypertension (ICD-9-CM codes 401–405), hyperlipidemia (ICD-9-CM code 272), chronic obstructive pulmonary disease (COPD) (ICD-9-CM codes 491, 492, 496), heart failure (ICD-9-CM codes 428), stroke (ICD-9-CM codes 430–438), and CAD (ICD-9-CM codes 410–414) were considered crucial factors affecting PAD occurrence.

### Statistical Analysis

We presented the mean and standard deviation for age and number and percentage for gender, age group, and comorbidities. The *t*-test and chi-square test were used to examine the distribution differences for continuous and categorical variables, respectively. We calculated the follow-up time in person-years to assess the incident density rates until the PAD was either identified or censored. We used univariable and multivariable Cox's proportional hazard regressions to investigate the association between PE and the risk of PAD over time, indicated by the hazard ratio (HR) with a 95% confidence interval (CI). The multivariable model was simultaneously adjusted for age, gender, and comorbidities of diabetes, hypertension, hyperlipidemia, COPD, heart failure, CAD, and stroke. In addition, after stratification based on gender, age, and follow-up time, the relative risk of PAD development in patients with PE was compared with that of the non-PE cohort. The comparative cumulative incidence of PAD between the PE and non-PE cohorts was assessed using the Kaplan–Meier method, and the differences were assessed using a log-rank test. All statistical analyses were performed using the SAS package (version 9.3 for Windows; SAS Institute, Inc., Cary, NC, USA). We adopted a two-tailed *P*-value of <0.05 as statistically significant.

## Results

**Figure 1:**
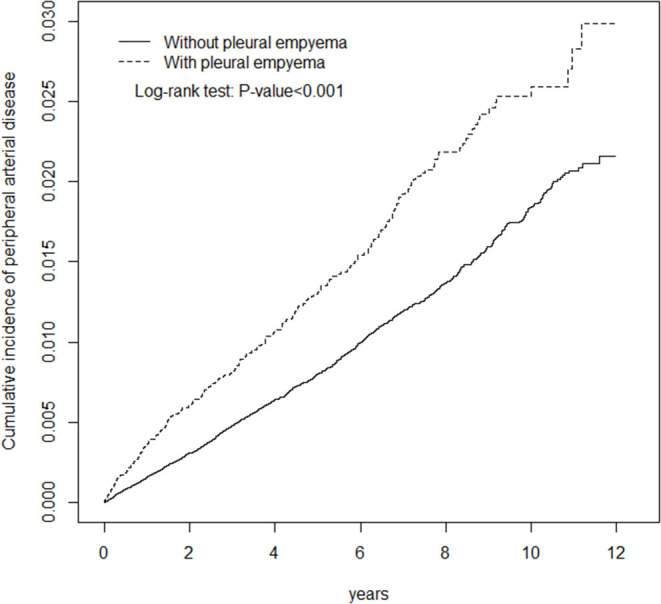
Cumulative incidence of peripheral arterial disease in patients with pleural empyema and comparison patients.

Table 1 presents the demographic characteristics and comorbidities of the study sample. This study had more male patients (77.2%), and 48.6% of the patients were of age more than 65 years (mean age 62.3 ± 16.8 years in the PE cohort and 61.5 ± 16.9 years in the non-PE cohort). Patients in the PE cohort exhibited a higher prevalence of comorbidities than those in the non-PE cohort (all *P* < 0.001). The average follow-up period was 3.49 years for the subjects with PE and 5.40 years for the non-PE cohort. The Kaplan–Meier analysis revealed that patients with PE had a significantly higher cumulative incidence of PAD than the non-PE controls (log-rank test, *P* < 0.001; Figure 1). The overall incidence of PAD was 61% higher in the PE cohort than in the non-PE cohort (2.76 vs. 1.72 per 1,000 person-years) with a crude HR of 1.61 (95% CI = 1.41–1.83) in the following 12 years (Table 2). After adjustment for age, gender, and comorbidities of diabetes, hypertension, hyperlipidemia, COPD, heart failure, CAD, and stroke, patients with PE were observed to be associated with an increased risk of PAD compared with those without PE [adjusted HR (aHR) = 1.18, 95% CI = 1.03–1.35]. The PAD incidence was higher with increased age and in those with comorbidities. Notably, compared with young adults (≤ 49 years of age), the aHR was 13.8 (95% CI = 10.5–18.2) for patients older than 75 years, 7.78 (95% CI = 5.98–10.1) for those aged 65–74 years, and 3.29 (95% CI 2.48–4.37) for those aged 50–64 years. Multivariate models revealed that PAD was independently associated with diabetes (aHR = 2.96, 95% CI = 2.61–3.35), hypertension (aHR = 1.49, 95% CI = 1.30–1.70), hyperlipidemia (aHR = 1.20, 95% CI = 1.00–1.44), heart failure (aHR = 1.82, 95% CI = 1.52–2.18), CAD (aHR = 1.39, 95% CI = 1.20–1.60), and stroke (aHR = 1.42, 95% CI = 1.24–1.64). The incidence of PAD was slightly higher in women than in men and increased with age in both cohorts (Table 3). A comparison between age-specific PE and non-PE cohorts revealed that the aHR of PAD was significantly higher in the ≤ 49 years age group (aHR = 5.34, 95% CI = 2.82–10.1). The incidence density rate of PAD was higher in the first 2 years, with an aHR of 1.35 (95% CI = 1.09–1.68) for patients with PE compared with those without PE.

**Table 1:** Comparisons in demographic characteristics and comorbidities in patients with and without pleural empyema.

	**Pleural empyema**		***P*-value**
	**No**	**Yes**	
	**(*N* =122,669)**	**(*N* =31,051)**	
Gender			0.91
Women	27,990(22.8)	7,076(22.8)	
Men	94,679(77.2)	23,975(77.2)	
Age stratified			0.69
≤ 49	31,854(26.0)	7,982(25.7)	
50–64	31,641(25.8)	7,986(25.7)	
65–74	40,121(32.7)	10,199(32.9)	
75+	19,053(15.5)	4,884(15.7)	
Age, mean ± SD^a^	61.5 ± 16.9	62.3 ± 16.8	<0.001
**Comorbidity**			
Diabetes	9,181(7.48)	9,440(30.4)	<0.001
Hypertension	18,594(15.2)	11,935(38.4)	<0.001
Hyperlipidemia	4,204(3.43)	2,246(7.23)	<0.001
COPD	5,698(4.65)	6,385(20.6)	<0.001
Heart failure	3,052(2.49)	3,259(10.5)	<0.001
CAD	9,220(7.52)	4,857(15.6)	<0.001
Stroke	8,527 (6.95)	6,847(22.1)	<0.001

Chi-square test,

a *t-test*.

**Table 2:** Incidence and hazard ratio for peripheral arterial disease and peripheral arterial disease-associated risk factor.

**Variable**	**Event**	**PY**	**Rate^**#**^**	**Crude HR (95% CI)**	**Adjusted HR†** **(95% CI)**
**Pleural empyema**					
No	1,140	662,279	1.72	1.00	1.00
Yes	299	108,518	2.76	1.61(1.41, 1.83)^***^	1.18(1.03, 1.35)^*^
**Age, year**					
≤ 49	62	233,569	0.27	1.00	1.00
50–64	224	213,823	1.05	3.99(3.01, 5.29)^***^	3.29(2.48, 4.37)^***^
65–74	725	244,851	2.96	11.4(8.83, 14.8)^***^	7.78(5.98, 10.1)^***^
75+	428	78,554	5.45	22.2(17.0, 29.1)^***^	13.8(10.5, 18.2)^***^
Gender					
Women	391	175,676	2.23	1.26(1.13, 1.42)^***^	1.04(0.92,1.17)
Men	1,048	595,121	1.76	1.00	1.00
**Comorbidity**					
Diabetes					
No	946	705,843	1.34	1.00	1.00
Yes	493	64,954	7.59	5.85(5.24, 6.52)^***^	2.96(2.61, 3.35)^***^
**Hypertension**					
No	815	663,572	1.23	1.00	1.00
Yes	624	107,225	5.82	4.96(4.46, 5.51)^***^	1.49(1.30, 1.70)^***^
**Hyperlipidemia**					
No	1,288	746,851	1.72	1.00	1.00
Yes	151	23,946	6.31	3.73(3.15, 4.41)^***^	1.20(1.00, 1.44)^*^
COPD					
No	1232	734,981	1.68	1.00	1.00
Yes	207	35,815	5.78	3.54(3.05, 4.11)^***^	1.11(0.95, 1.31)
**Heart failure**					
No	1,268	754,791	1.68	1.00	1.00
Yes	171	16,006	10.7	6.58(5.60, 7.73)^***^	1.82(1.52, 2.18)^***^
**CAD**					
No	1,096	721,861	1.52	1.00	1.00
Yes	343	48,936	7.01	4.74(4.19, 5.35)^***^	1.39(1.20, 1.60)^***^
**Stroke**					
No	1,120	724,096	1.55	1.00	1.00
Yes	319	46,701	6.83	4.59(4.04, 5.20)^***^	1.42(1.24, 1.64)^***^

# Rate, incidence rate, per 1,000 person-years.

Crude HR, relative hazard ratio; ^†^adjusted HR: multiple analysis including age, gender, and comorbidities of diabetes, hypertension, hyperlipidemia, COPD, heart failure, CAD, and stroke;

* p < 0.05,

*** *p < 0.001*.

**Table 3:** Comparison of incidence densities of peripheral arterial disease hazard ratio between with and without pleural empyema by gender, age, and follow-up time.

	**Pleural empyema**							
	**No**			**Yes**				
	**Event**	**PY**	**Rate^**#**^**	**Event**	**PY**	**Rate^**#**^**	Crude HR^*^	**Adjusted HR†**
							**(95% CI)**	**(95% CI)**
**Gender**								
Women	304	151,837	2.00	87	23,838	3.65	1.83(1.44, 2.33)^***^	1.23(0.95, 1.59)
Men	836	510,442	1.64	212	84,679	2.50	1.53(1.32, 1.78)^***^	1.15(0.98, 1.35)
**Stratify age**								
≤ 49	17	193,683	0.09	45	39,885	1.13	12.9(7.37, 22.5)^***^	5.34(2.82, 10.1)^***^
50–64	146	181,956	0.80	78	31,867	2.45	3.17(2.41, 4.17)^***^	1.04(0.76, 1.43)
65–74	596	215,454	2.77	129	29,397	4.39	1.65(1.36, 2.00)^***^	0.89(0.73, 1.09)
75+	381	71,186	5.35	47	7,368	6.38	1.20(0.89, 1.63)	0.88(0.64, 1.21)
**Follow-up time, years**								
≤ 2	357	231,384	1.54	137	43,486	3.15	2.02(1.66, 2.46)^***^	1.35(1.09, 1.68)^**^
>2	783	430,894	1.82	162	65,032	2.49	1.38(1.17, 1.63)^***^	1.07(0.89, 1.28)

# Rate, incidence rate, per 1,000 person-years;

* Crude HR, relative hazard ratio; ^†^adjusted HR: multiple analysis including age, gender, and comorbidities of diabetes, hypertension, hyperlipidemia, COPD, heart failure, CAD, and stroke;

** p < 0.01,

*** *p < 0.001*.

## Discussion

To the best of our knowledge, this is the first nationwide retrospective cohort study to evaluate the PAD risk associated with PE. Notably, patients with PE had a 1.18-fold higher risk of PAD than those without PE. The PAD incidence increased with increasing age, when there are comorbidities, and when there was no gender difference. Compared with previous studies, PAD exhibited female sex preponderance (19). Our study revealed notable results that were different from those of previous studies on PE or PAD. Our study observed an increased risk of PAD if patients with PE were younger than 49 years and within the 2-year period of PE. Some possible mechanisms might explain our results. Ethnicity or genetics could have played a crucial role because PAD was more common in African Americans from the report of the National Health and Nutrition Examination Survey (19). Comorbidities were observed in both PE and PAD. Diabetes could cause the risk of not only *Klebsiella pneumoniae*–related PE but also atherosclerosis (20). A 5-year study indicated that the most common pathogens of PE were *K. pneumoniae*, followed by *Escherichia coli, Proteus mirabilis*, and others (21). A report regarding chest infections in Japan revealed that the percentages of lung abscesses and empyema were higher in patients infected with *Klebsiella* species and having diabetes and a smoking history (22). Furthermore, another common pathogen causing PE was indicated to be *Streptococcus pneumoniae* and was reported to intensify atherosclerosis and contribute to PAD in patients with PE (23). Smoking caused deleterious effects on cardiovascular and pulmonary systems, impeding the functional examinations, such as forced vital capacity, forced expiratory volume in 1 s, intima-media thickness, and ankle-brachial pressure index (ABI) (24), and lower ABI helps diagnose PAD (19). Due to data restriction and limitation, our study could not collect the smoking habit for the analysis of smoking, PE, and PAD, but we used COPD to represent smoking-related diseases and to reduce the confounding bias. Moreover, a Korean study indicated that the young age of diabetes onset was a distinctive feature of Asians (25). This finding supports our result that young people with PE might develop PAD. In addition, previous studies have reported the association between other comorbidities and PE (14, 15). Notably, patients with CVD, stroke, hyperlipidemia, and diabetes had an identically higher risk of generalized atherosclerosis with PAD. Moreover, age, hypertension, and hyperlipidemia were considered risk factors for PAD. Pleural effusion was most often caused by heart failure, cancer, and pneumonia and could be a potential risk factor for PE (26). Patients with heart failure and reduced ejection fraction had a higher prevalence of PAD (27). PAD can manifest in various ways, such as having no signs and symptoms or having intermittent claudication, ischemic ulcers, and gangrene. Limb loss with amputation was more prevalent in patients with ABI <0.5 (28). Moreover, PE could be associated with infection susceptibility and prolonged hospitalization. Of course, we just emphasized the association between PAD and PE. Through our big data analysis, we found the result. Further basic research should be done in the future such as gene analysis. In our personal opinion, inflammation whether caused by infection or other chronic disease is one of the possible explanations.

Notably, being the first cohort study focusing on PAD incidence in patients with PE and using a large sample size for meaningful analyses were our study's strengths. Nonetheless, our study had several limitations. First, we could not entirely avoid the confounding effects of the preexisting comorbidities of PAD such as all cancers and inflammatory diseases, and any deaths from PAD must have been excluded. Second, NHIRD data on environmental risk factors influencing PAD and PE, such as cigarette smoking, alcohol consumption, nutritional status indicators such as body mass index, and family history, could not be obtained. Third, the laboratory data, such as ABI, chest X-ray images, bacterial species culture such as Streptococcus or *Klebsiella* species or Gram stain, and blood glucose level could not be obtained. Finally, even though our study revealed the possible role of PE in increasing the risk of PAD, further research is warranted to unravel the pathogenicity mechanisms involved in the development of PAD in patients with PE.

## Conclusion

Our analysis determined that patients with PE had an increased risk of PAD, especially if they were younger than 49 years and within the 2-year diagnosis of PE.

## Data Availability Statement

The datasets presented in this article are not readily available because the dataset used in this study is held by the Taiwan Ministry of Health and Welfare (MOHW). The Ministry of Health and Welfare must approve our application to access this data. Any researcher interested in accessing this dataset can submit an application form to the Ministry of Health and Welfare requesting access. Please contact the staff of MOHW (Email: stcarolwu@mohw.gov.tw) for further assistance. Taiwan Ministry of Health and Welfare Address: No.488, Sec. 6, Zhongxiao E. Rd., Nangang Dist., Taipei City 115, Taiwan (R.O.C.). Phone: +886-2-8590-6848. All relevant data are within the paper. Requests to access the datasets should be directed to stcarolwu@mohw.gov.tw.

## Ethics Statement

The studies involving human participants were reviewed and approved by This study was approved by the Institutional Review Board of China Medical University (CMUH104-REC2-115-AR4). Written informed consent for participation was not required for this study in accordance with the national legislation and the institutional requirements.

## Author Contributions

H-HC and C-HK: conception and design. C-HK: provision of study materials. All authors: collection and/or assembly of data, data analysis and interpretation, manuscript writing, and final approval of the manuscript. All authors have contributed significantly and agree with the manuscript content.

## Conflict of Interest

The authors declare that the research was conducted in the absence of any commercial or financial relationships that could be construed as a potential conflict of interest.
